# MR imaging by 3D T1-weighted black blood sequences may improve delineation of therapy-naive high-grade gliomas

**DOI:** 10.1007/s00330-020-07314-6

**Published:** 2020-10-10

**Authors:** Tom Finck, Jens Gempt, Claus Zimmer, Jan S. Kirschke, Nico Sollmann

**Affiliations:** 1 Department of Diagnostic and Interventional Neuroradiology, Klinikum rechts der Isar, Technische Universität München, Ismaninger Str. 22, 81675 Munich, Germany; 2 Department of Neurosurgery, Klinikum rechts der Isar, Technische Universität München, Ismaninger Str. 22, 81675 Munich, Germany; 3 TUM-Neuroimaging Center, Klinikum rechts der Isar, Technische Universität München, Munich, Germany

**Keywords:** Brain neoplasms, Blood-brain barrier, Glioma, Magnetic resonance imaging

## Abstract

**Objectives:**

To investigate the value of contrast-enhanced (CE) turbo spin echo black blood (BB) sequences for imaging of therapy-naive high-grade gliomas (HGGs).

**Methods:**

Consecutive patients with histopathologically confirmed World Health Organization (WHO) grade III or IV gliomas and no oncological treatment prior to index imaging (March 2019 to January 2020) were retrospectively included. Magnetic resonance imaging (MRI) at 3 Tesla comprised CE BB and CE turbo field echo (TFE) sequences. The lack/presence of tumor-related contrast enhancement and satellite lesions were evaluated by two readers. Sharper delineation of tumor boundaries (1, bad; 2, intermediate; 3, good delineation) and vaster expansion of HGGs into the adjacent brain parenchyma on CE BB imaging were the endpoints. Furthermore, contrast-to-noise ratios (CNRs) were calculated and compared between sequences.

**Results:**

Fifty-four patients were included (mean age: 61.2 ± 15.9 years, 64% male). The vast majority of HGGs (51/54) showed contrast enhancement in both sequences, while two HGGs as well as one of six detected satellite lesions were depicted in CE BB imaging only. Tumor boundaries were significantly sharper (R1: 2.43 ± 0.71 vs. 2.73 ± 0.62, *p* < 0.001; R2: 2.44 ± 0.74 vs. 2.77 ± 0.60, *p* = 0.001), while the spread of HGGs into the adjacent parenchyma was larger when considering CE BB sequences according to both readers (larger spread in CE BB sequences: R1: 23 patients; R2: 20 patients). The CNR for CE BB sequences significantly exceeded that of CE TFE sequences (43.4 ± 27.1 vs. 32.5 ± 25.0, *p* = 0.0028).

**Conclusions:**

Our findings suggest that BB imaging may considerably improve delineation of therapy-naive HGGs when compared with established TFE imaging. Thus, CE BB sequences might supplement MRI protocols for brain tumors.

**Key Points:**

*• This study investigated contrast-enhanced (CE) T1-weighted black blood (BB) sequences for improved MRI in patients with therapy-naive high-grade gliomas (HGGs).*

*• Compared with conventionally used turbo field echo (TFE) sequences, CE BB sequences depicted tumor boundaries and spread of HGGs into adjacent parenchyma considerably better, which also showed higher CNRs.*

*• Two enhancing tumor masses and one satellite lesion were exclusively identified in CE BB sequences, but remained undetected in conventionally used CE TFE sequences.*

## Introduction

Globally, over 300,000 patients are affected by malignant primary brain tumors each year [1, 2]. Although this accounts for only about 2% of all cancers, mortality in affected patients is exceedingly high. As such, the median survival times for glioblastoma multiforme and anaplastic astrocytoma, the most common entities of primary brain tumors and categorized as high-grade glioma (HGG), lie at only 14 and 16 months on average [2–4].

While therapy relies on factors such as tumor grade and functional eloquence of the affected brain region, the most commonly used scheme for treatment of HGGs includes debulking surgery and extended focal irradiation, as well as adjuvant chemotherapy [5–8]. Unfortunately, although this aggressive regime is routinely used in the clinical setting, prognosis of affected patients has remained largely stagnant for the last decades. In spite of the overall poor prognosis, factors that impair (i.e., higher grade of the tumor) or favor outcomes (i.e., complete surgical resection) have nonetheless been identified and are of considerable clinical relevance [8–11].

The aggressive biological behavior of HGGs often goes hand-in-hand with destruction of the blood-brain barrier and consecutive contrast enhancement in T1-weighted magnetic resonance imaging (MRI), highlighting the usefulness of post-gadolinium MRI in neuro-oncological imaging [12, 13]. Both the clear depiction of contrast-enhancing tumor boundaries and the lack or presence of satellite lesions are paramount to presurgical planning of HGG resections and prognostically relevant as incomplete resection is correlated to lower survival rates and quality of life [10, 14–18].

While conventional turbo field echo (TFE) imaging before and after contrast administration has been long established to assess tumor-related contrast enhancement and spread, recent studies have hinted at the value of advanced T1-weighted sequences for the depiction of intracranial contrast-enhancing pathology [19, 20]. Although initially developed for vessel wall imaging, T1-weighted black blood (BB) sequences in particular have gained attention for depicting intracranial malignancies. Recently, MRI protocols making use of contrast-enhanced (CE) BB sequences were able to detect significantly more cerebral metastases and had a higher sensitivity to identify leptomeningeosis carcinomatosa compared with more established T1-weighted sequences for this purpose (e.g., gradient echo and spin echo sequences) [19, 21]. Nevertheless, data on the utility and potential superiority of this novel sequence in a homogeneous patient cohort with the diagnosis of HGGs are still lacking.

Therefore, we strive to investigate the use of CE BB imaging in delineating HGGs and to compare its performance with that of the established CE TFE sequences. We hypothesize that sharper delineation of tumor boundaries, vaster expansion into the adjacent brain parenchyma, and improved detection of satellite lesions may be achieved with CE BB imaging.

## Materials and methods

### Study protocol

This study represents a retrospective analysis of prospectively collected monocentric data. Approval of the Institutional Review Board (registration number: 340/16 S) and written informed consent were obtained for the prospective data collection. Acquisition of non-CE and CE BB sequences was performed as part of the clinical routine, with BB imaging being implemented in the default glioma imaging protocol at our institution since early 2019.

For patient inclusion, the hospital-intern Picture Archiving and Communication System (PACS) database was retrospectively searched from March 2019 to January 2020 considering the following inclusion criteria: (1) acquisition of TFE and BB sequences before and after administration of a contrast agent in the same imaging session (as part of a standardized brain tumor imaging protocol); (2) presence of an intracranial neoplastic mass (supratentorial and/or infratentorial) according to imaging; (3) histopathological confirmation of a HGG (using tissue analysis of probes derived from biopsy or harvested during tumor resection); and (4) age above 18 years. The following exclusion criteria were defined: (1) previous debulking surgery (performed before the time point of imaging considered in this study); (2) previous oncological therapy (received before the time point of imaging considered in this study); (3) severe motion artifacts in imaging data not allowing for diagnostic use; and (4) artifacts in imaging data stemming from foreign bodies or implants (e.g., ventriculo-peritoneal shunts).

### MRI protocol

MRI data were acquired following a standardized, institute-specific multi-sequence protocol that remained the same during the time interval of study inclusion. Two 3-Tesla scanners (Achieva dStream and Ingenia, Philips Healthcare) with a 32-channel head coil were available for image acquisitions.

Scan parameters were the same in all patients for the TFE sequences (TR = 9 ms; TE = 4 ms; flip angle = 8°; acquisition mode, 3D; acquisition duration, 2:26 min; acquired in the sagittal plane with isotropic voxel size of 1 mm^3^) and BB sequences (turbo spin echo; TR = 4000 ms; TE = 35 ms; flip angle = 90°; acquisition mode, 3D; acquisition duration, 1:36 min; acquired in the sagittal plane with isotropic voxel size of 1 mm^3^), with acquisitions of these sequences being performed before and after intravenous administration of a contrast agent in the same imaging session.

A dose of 0.2 ml per kg body weight of gadoteric acid (DOTAGRAF® 0.5 mmol/ml, Jenapharm GmbH) was administered automatically via a pressure pump. CE TFE imaging was acquired ~ 4 min and CE BB imaging ~ 7 min after contrast injection.

### Image analysis

Two neuroradiologists with 3 years (reader 1 = R1) and 7 years of experience (reader 2 = R2) in neuroradiological imaging analyzed the images on separate PACS working stations (IDS7, Sectra AB) independently from each other. Investigators were blinded regarding the final histopathological diagnosis but could not be reliably blinded to the sequence type due to the distinct appearance of both sequences (TFE or BB imaging).

CE TFE and CE BB sequences were evaluated in all patients, with the order of cases being subject to randomization. Non-CE TFE and non-CE BB as well as fluid-attenuated inversion recovery (FLAIR) sequences of the same imaging session were available for co-registration; other sequences were not provided for evaluation (e.g., diffusion-weighted or perfusion-weighted sequences). The investigators were allowed to use axial, sagittal, and coronal slices and to manually adapt image contrast at the working stations.

The lack or presence of contrast-enhancing HGGs (only visible in CE TFE, only visible in CE BB, or visible in both sequences), as well as the lack or presence of satellite lesions (only visible in CE TFE, only visible in CE BB, or visible in both sequences) of the primary tumor mass were assessed. Satellite lesions were defined as contrast-enhancing lesions that were located clearly outside of the perifocal hyperintensities of FLAIR sequences that surrounded the main bulk of the HGGs. For each contrast-enhancing HGG, binary determination was made if the spreading into the adjacent brain parenchyma, i.e., the extent of contrast enhancement, was comparable or greater in one of both investigated sequences (more extensive in CE TFE, more extensive in CE BB, or same extent in both sequences).

Overall image quality was rated on a 5-point Likert scale (1, bad; 2, medium to bad; 3, medium; 4, good; and 5, very good image quality). Delineation of contrast enhancement of the HGGs against surrounding brain parenchyma as well as conspicuity of arterial blood vessels of the Circulus of Willisii was rated on 3-point Likert scales (1, bad; 2, intermediate; and 3, good delineation/conspicuity).

For quantitative image analysis, the contrast-to-noise ratio (CNR) was estimated as follows on CE TFE and CE BB sequences [22]:

$$ \mathrm{CNR}=\left({\mathrm{Signal}\ \mathrm{Intensity}}_{\mathrm{Lesion}}\hbox{--} {\mathrm{Signal}\ \mathrm{Intensity}}_{\mathrm{NAWM}}\right)/{\mathrm{Standard}\ \mathrm{Deviation}}_{\mathrm{NAWM}} $$

To calculate the CNR, a representative region of interest (ROI) of 8 mm in diameter was manually drawn in the contrast-enhancing part of the HGG, with an equally sized ROI being placed in the normal-appearing white matter (NAWM) of the contralateral hemisphere. The quantitative image analysis was performed by R1.

### Statistical analysis

GraphPad Prism (version 8.3.1, GraphPad Software Inc.) was used for statistical analysis. Values are given as counts or mean ± standard deviation and ranges. The Kolmogorov-Smirnov test indicated non-Gaussian data distribution for image quality, delineation of HGGs to surrounding brain parenchyma, conspicuity of blood vessels, and Gaussian data distribution for CNRs.

The Wilcoxon matched pair signed-rank test was used for significance testing between scores assigned for image quality and delineation of HGGs to surrounding brain parenchyma, as well as for conspicuity of blood vessels. Paired *t*-tests were used for significance testing for CNRs between both sequences. *P* values < 0.05 were considered statistically significant, with adjustment for multiple testing being performed using the Benjamini-Hochberg procedure with a false discovery rate of 25%.

Inter-reader agreement was assessed with the intraclass correlation coefficient (ICC) and related 95% confidence intervals (CIs), using single measurements for absolute agreement in a two-way random model. Sharper delineation of HGGs against the surrounding parenchyma, as well as a vaster spread of contrast-enhancing HGGs into the adjacent brain parenchyma, were defined as the study’s endpoints.

## Results

### Patient characteristics

From the 501 patients that were retrieved, 447 did not meet the abovementioned inclusion criteria. Fifty-four patients (64% male) at a mean age of 61.2 ± 15.9 years (age range: 22–86 years) could be included. Histopathology reports of the surgical probes revealed glioma grade IV (glioblastoma multiforme) in 44 patients and glioma grade III (anaplastic astrocytoma) in 10 patients according to the grading of the World Health Organization (WHO).

### Image quality and tumor delineation

Comparable good or very good image quality was noted in CE TFE and CE BB sequences for R1 (4.21 ± 0.99 vs. 4.34 ± 0.90, *p* = 0.45) and R2 (4.04 ± 0.95 vs. 4.16 ± 0.71, *p* = 0.43). Clearer delineation of the contrast-enhancing HGGs was achieved when considering the CE BB images compared with CE TFE images according to R1 (2.43 ± 0.71 vs. 2.73 ± 0.62, *p* < 0.001) and R2 (2.44 ± 0.74 vs. 2.77 ± 0.60, *p* = 0.001).

Vessels could be sharply delineated from the surrounding brain parenchyma in both sequences without a significant difference (R1 and R2, *p* > 0.05). The results on vessel delineation as well as image quality and delineation of tumor boundaries for both readers are given in Table 1.

**Table 1:** Ratings for both readers (R1 and R2) on image quality, delineation of tumor against the surrounding brain parenchyma, and differentiation of vessels against the surrounding brain parenchyma on contrast-enhanced (CE) turbo field echo (TFE) and CE black blood (BB) imaging

	CE TFE	CE BB	*p*
Image quality R1	4.21 ± 0.99	4.34 ± 0.90	0.45
Image quality R2	4.04 ± 0.95	4.16 ± 0.71	0.43
Tumor delineation R1	2.43 ± 0.71	2.73 ± 0.62	< 0.001
Tumor delineation R2	2.44 ± 0.74	2.77 ± 0.60	0.001
Vessel differentiation R1	2.81 ± 0.39	2.81 ± 0.45	0.99
Vessel differentiation R2	2.94 ± 0.25	2.90 ± 0.31	0.50

### Enhancement characteristics

Overall, 51 out of 54 HGGs showed contrast enhancement in both sequences, while one HGG did not show contrast enhancement according to both readers. However, contrast enhancement of two HGGs was only detected on CE BB imaging and therefore missed on CE TFE imaging according to both readers. There were no HGGs detectable in CE TFE that would not have been detected in CE BB imaging (Table 2).

**Table 2:** Enhancement characteristics according to both readers (R1 and R2) of the primary high-grade gliomas (HGGs) and satellite lesions (if applicable) for contrast-enhanced (CE) turbo field echo (TFE) and CE black blood (BB) imaging. In total, 54 patients with HGGs were included, while 53 HGGs showed contrast enhancement in at least one of those sequences (one HGG did not show contrast enhancement in CE TFE and CE BB imaging)

HGG	R1	R2
Enhancement visible in CE TFE and CE BB sequences	51	51
No contrast enhancement	1	1
Enhancement only visible in CE BB sequences	2	2
Enhancement only visible in CE TFE sequences	0	0
Satellite lesions	R1	R2
Enhancement visible in CE TFE and CE BB sequences	5	4
No satellite lesions detectable	48	49
Enhancement only visible in CE BB sequences	1	1
Enhancement only visible in CE TFE sequences	0	0

**Fig. 1:**
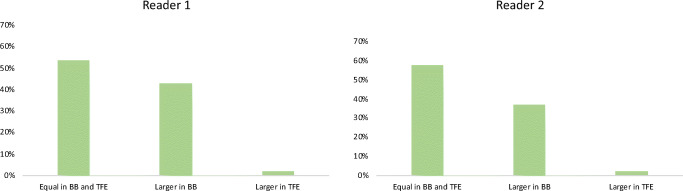
Bar chart indicating the spread of contrast-enhancing high-grade gliomas (HGGs) as evaluated by visual grading. Notable is the higher fraction of patients with more extensive HGG spread when considering the contrast-enhanced (CE) black blood (BB) sequences in comparison with CE turbo field echo (TFE) sequences

The primary endpoint of larger discernible contrast enhancement of HGGs was reached more often for the CE BB than the CE TFE sequences for R1 (23 patients vs. 1 patient) and R2 (20 patients vs. 2 patients), as depicted in Fig. 1 and Table 3. Beyond the improved delineation of contrast enhancement of HGGs, one of the six satellite lesions detected by R1 (five satellite lesions detected by R2) was detectable in CE BB only (Table 2). Quantitative measurements of the contrast-enhancing parts of the HGGs revealed a superior CNR of CE BB compared with CE TFE sequences (43.4 ± 27.1 vs. 32.5 ± 25.0, *p* = 0.0028).

**Table 3:** Results of the assessment of both readers (R1 and R2) regarding tumor spread into the adjacent brain parenchyma, which can be equal for both sequences or more extensive in contrast-enhanced (CE) turbo field echo (TFE) or CE black blood (BB) imaging. In total, 54 patients with high-grade gliomas (HGGs) were included, while 53 HGGs showed contrast enhancement in at least one of those sequences (one HGG did not show contrast enhancement in CE TFE and CE BB imaging)

Spread of enhancement	R1	R2
Equal in CE TFE and CE BB sequences	29	31
Larger in CE BB sequences	23	20
Larger in CE TFE sequences	1	2

**Fig. 2:**
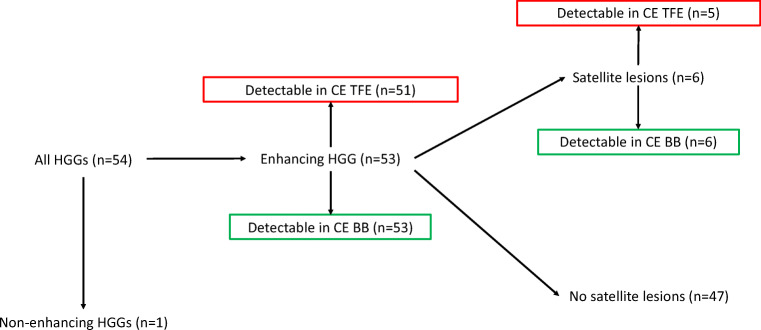
Flowchart showing the patient numbers and contrast enhancement characteristics of high-grade gliomas (HGGs) according to contrast-enhanced (CE) turbo field echo (TFE) and CE black blood (BB) imaging

**Fig. 3:**
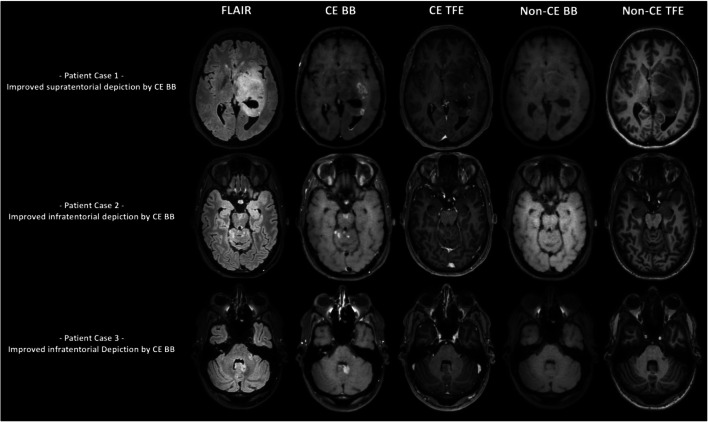
Exemplary cases for better depiction of contrast-enhancing high-grade gliomas (HGGs) in contrast-enhanced (CE) black blood (BB) imaging versus CE turbo field echo (TFE) imaging

**Fig. 4:**
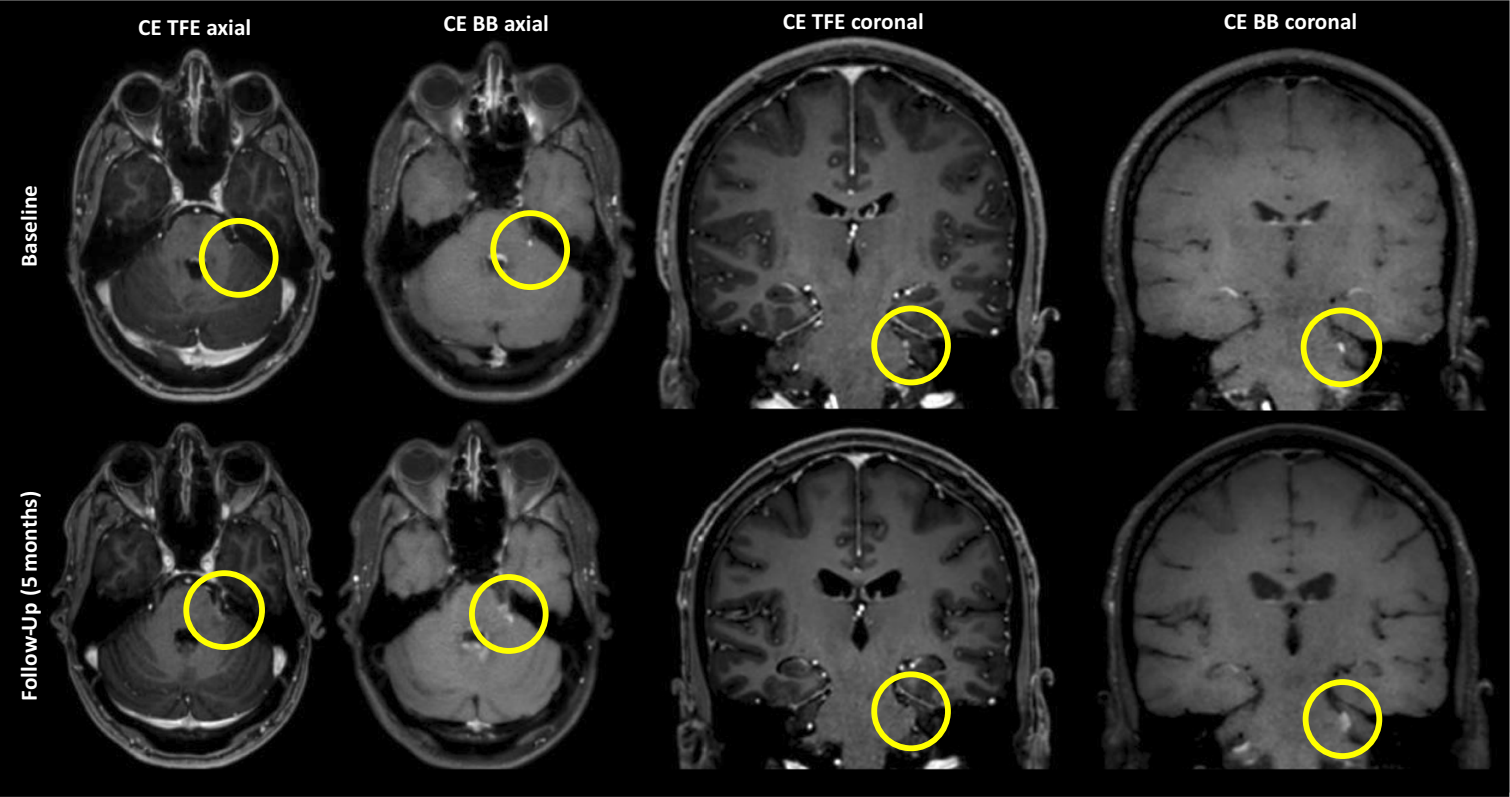
Follow-up imaging in an exemplary patient case with clearly depictable spread of a high-grade glioma (HGG) in the posterior fossa on 5-month follow-up imaging that was clearly visualized in contrast-enhanced (CE) black blood (BB) sequences, but not or only marginally depicted on CE turbo field echo (TFE) sequences at baseline (yellow circles)

A flowchart illustrating how contrast-enhancing tumors were identified in both sequences is given in Fig. 2. Illustrative cases for the improved identification of contrast-enhancing HGGs in different brain regions are provided by Fig. 3. Furthermore, to further elucidate CE BB sequences, we provide the therapy-naive baseline and follow-up imaging of an exemplary patient case with a multifocal glioblastoma multiforme that was in part initially only detectable on CE BB imaging (Fig. 4). Follow-up MRI after approximately 5 months and interim radio-chemotherapy reveals an inhomogeneous spread of the initially suspected contrast-enhancing multifocal HGG, compatible with the growth pattern of a non-responsive tumor.

### Inter-reader reliability

Consistent readings were given by both readers, as reflected by an ICC that ranged from 0.75 (95%-CI 0.56, 0.80) for the image quality of CE BB to excellent values of 0.94 (95%-CI 0.90, 0.97) for the delineation of contrast-enhancing HGGs against surrounding brain parenchyma in CE BB sequences. Detailed values for the inter-reader ICC are given in Table 4.

**Table 4:** Intraclass correlation coefficients (ICCs, absolute agreement in a two-way random model) and corresponding 95% confidence intervals (CIs) between both readers (R1 and R2) for image quality and delineation of tumor tissue to adjacent brain parenchyma in contrast-enhanced (CE) turbo field echo (TFE) and CE black blood (BB) imaging

Inter-reader agreement	
ICC image quality CE TFE sequences	0.91 (95%-CI 0.84, 0.95)
ICC image quality CE BB sequences	0.75 (95%-CI 0.56, 0.80)
ICC delineation tumor CE TFE sequences	0.90 (95%-CI 0.82, 0.94)
ICC delineation tumor CE BB sequences	0.94 (95%-CI 0.90, 0.97)

## Discussion

In an effort to improve neuro-oncological imaging, we evaluated whether the previously reported increased sensitivity of CE BB compared with that of conventional CE T1-weighted sequences (e.g., gradient echo and spin echo sequences) in depicting metastatic brain lesions could be replicated for therapy-naive HGGs. Our main findings were as follows: (i) in a substantial proportion of patients, the spread of contrast-enhancing HGGs appeared larger in CE BB than in CE TFE imaging (Table 3); (ii) superior CNRs as well as significantly sharper delineation of HGGs against the surrounding brain parenchyma are achievable when utilizing CE BB imaging (Table 1); and (iii) in some cases, satellite lesions and contrast enhancement of HGGs appear to be missed when CE BB imaging is not part of the MRI protocol (Table 2).

Imaging in neuro-oncology has undergone an extensive evolution from the first ever depiction of intracranial masses with X-ray through the establishment of CE MRI in the 1980s [12, 13, 23]. Today, there is strong evidence that contrast-enhancing tumor volume and extent on baseline MRI scans is a prognostic factor for morbidity and overall survival in HGGs [10, 14–18]. The status of MRI as the first-line method for resection planning therefore relies on availability of the imaging technique that best reflects the in-situ morphology of the suspected malignancy. With this in mind, the overwhelming share of patients that had larger areas of contrast-enhancing HGGs on CE BB compared with CE TFE sequences in the present study is worthy of mention and raises the question if—and how much—information gets “lost” when solely relying on conventional CE TFE imaging.

However, TFE sequences before and after contrast administration reflect the standard T1-weighted sequences in the clinical setting for imaging of brain tumors in most centers dealing with neuro-oncological imaging. Our results probably allow for the assumption that resection of HGGs could be more incomplete when CE BB imaging is not consulted for surgical planning in the preoperative workup of patients suffering from HGGs. Even if proof can only be provided via histopathological sampling, the time-dependent changes that we exemplarily give (Fig. 4) reinforce the assumption that while both sequences allow identifying an impaired blood-brain barrier, CE BB imaging might be more sensitive and can potentially depict earlier stages of blood-brain barrier breakdown and disease.

Although not even the most aggressive debulking surgery can be considered curative in HGGs, there is abundant data confirming that incomplete resection of contrast-enhancing tumors correlates to reduced overall survival and can have negative impact on the quality of life in affected patients, thus clearly favoring complete extent of resection [10, 14–18]. Furthermore, it is known that a vast majority of HGG relapses grow from within the tissue adjacent to the resection border [24]. In addition, a tendency of growth toward functionally eloquent brain structures has been suggested [25]. Hence, presurgical planning on the most sensitive CE MRI sequence possible becomes crucially important. In the backlight of this, it seems clinically relevant that contrast enhancement in two HGGs as well as one contrast-enhancing satellite lesion in our patient cohort would not have been unequivocally detected with a conventional brain tumor imaging protocol that does not include BB imaging. This is coherent to our superior measurements of CNRs in CE BB sequences and in agreement with previous studies that reported superior detectability of contrast-enhancing multiple sclerosis lesions, heterogenous groups of primary or secondary brain tumors, and leptomeningeosis carcinomatosa in CE BB imaging [20, 21]. As the abovementioned satellite lesion was located distant (outside of the perifocal FLAIR edema/infiltration zone) of the main HGG bulk, it becomes obvious that the affected patient would most likely have been insufficiently treated (surgically and radio-oncologically) without CE BB sequences and would potentially have suffered from a worsened prognosis.

Balancing tumor resection with the risk of treatment-related neurological morbidity is a priority for neurosurgeons in brain tumor surgery [26, 27]. Beyond the possibility of performing protective, yet time-intensive tests such as cortical stimulation mapping during surgery, a clear definition of HGG boundaries on planning MRI is key to safely perform navigated excision. With this in mind, the sharper delineation of HGGs in CE BB versus CE TFE imaging seems of special interest. Specifically, evaluations of both readers showed good identification of contrast-enhancing HGG borders in CE BB with only intermediate differentiation in CE TFE sequences. Especially adjacent to eloquent brain structures, such as the Broca’s area or the primary motor cortex, a clearer delineation may provide the required certainty to prevent too aggressive resections causing functional deterioration and unnecessary treatment-related morbidity. Objections on the risk of confounding tumor tissue with saturated vessel signals on CE BB sequences seem unwarranted as vessels were reliably identifiable for both sequences, yet with different characteristic appearances.

While the inter-reader agreement was excellent when it comes to HGG delineation from the surrounding brain parenchyma, lower values were, however, noted for image quality of CE BB sequences. This finding needs to be addressed given that a reliable diagnostic tool must deliver reproducible results. However, given the novelty of the sequence being part of the glioma imaging protocol at our institution, it seems reasonable that neuroradiologists first have to get used to the image impression and quality prior to achieving high correlations between individual readings.

Implementation of new MRI sequences, such as the proposed BB sequence for HGG imaging, is not the only path to achieve superior conspicuity of pathologies with an impaired blood-brain barrier. Generally speaking, elevating the CNR by increasing the dose of applied contrast agent and performing standard T1-weighted post-contrast sequences would be a much more straightforward and common approach, thus not requiring clinicians to familiarize with newly developed sequence protocols. Improved depiction of intracranial metastases with double-dose contrast imaging has thus already been suggested, and it led to the assumption that a higher dosage of gadolinium might be well suitable for neuro-oncological imaging [28, 29]. On the other hand, data has since then accumulated that highlight the dose-dependent deposition of gadolinium in various tissues and fed a growing controversy that resulted in a more restrictive use of contrast agents for MRI [30]. As a consequence, many research efforts have ever since focused on implementing methods to reduce gadolinium dosage without impairing image sensitivity. Even if we are still in the nascent days of improving medical imaging through artificial intelligence, deep learning methods, such as generative adversarial networks, can already reliably boost the enhancement pattern of a low-dose MRI exam and thus simulate a full-dose scan [31]. To further promote such evolutions, providing the MRI sequence with the highest sensitivity to detect pathology is of paramount importance. Results of this study may not only improve neuro-oncological imaging in the direct clinical setting, but could also pave the way for a next iteration of artificial intelligence approaches that might have the potential to further wean our dependency on gadolinium.

Some limitations of this study need to be mentioned. First, the retrospective character of the underlying analysis makes it speculative to predict surgical outcomes based on individual sequences from a complete MRI study. Second, due to the standardized MRI protocol, CE BB imaging was always acquired ~ 3 min after CE TFE imaging. This might seem problematic as numerous studies have hinted at the superior depiction of contrast-enhancing intracranial malignancies in T1-weighted scans with delayed enhancement. While we are aware of this potential bias in our study protocol, we nonetheless note that the mere difference of approximately three minutes in sequence acquisitions is far less than the delayed acquisitions in studies focusing on brain metastases, and the underlying cause of an impaired blood-brain barrier and its consequences for contrast enhancement characteristics might be extrapolated to other malignant tissues such as HGGs (range in delay from 10 to 75 min) [32, 33]. We further acquired a preliminary sample of later-phase CE TFE and CE BB images after the here-investigated standard CE sequences for a subset of five patients with therapy-naive HGGs and were not able to detect any significant increases in CNR after the extra delay of about four minutes. Hence, we presume that the effect of timing in the enhancement characteristics in CE BB versus CE TFE imaging can be considered marginal at most. Third, the evaluations of tumor boundaries and size have been made qualitatively due to the very heterogeneous HGG shapes that did not allow for reliable quantitative segmentation. This approach, however, resembles daily clinical image reading where most diagnostic evaluation is still based on visual assessment.

In conclusion, we report the first evaluation of a CE BB sequence in therapy-naive imaging of HGGs. Our findings demonstrate superior tumor delineation against the surrounding brain parenchyma, a wider spread of contrast-enhancing parts of the HGG, and the ability to depict more satellite lesions when compared with established CE TFE imaging. Albeit necessitating confirmation by larger prospective studies with location-dependent histopathological confirmation, these observations advocate for a relevant role of CE BB as a first-line MRI sequence in neuro-oncological imaging.

## Abbreviations

BB: Black blood
CE: Contrast-enhanced
CI: Confidence interval
CNR: Contrast-to-noise ratio
FLAIR: Fluid-attenuated inversion recovery
HGG: High-grade glioma
ICC: Intraclass correlation coefficient
MRI: Magnetic resonance imaging
NAWM: Normal-appearing white matter
PACS: Picture Archiving and Communication System
R1: Reader 1
R2: Reader 2
ROI: Region of interest
TFE: Turbo field echo
WHO: World Health Organization
